# Biofilm marker discovery with cloud-based dockerized metagenomics analysis of microbial communities

**DOI:** 10.1093/bib/bbae429

**Published:** 2024-09-11

**Authors:** Etienne Z Gnimpieba, Timothy W Hartman, Tuyen Do, Jessica Zylla, Shiva Aryal, Samuel J Haas, Diing D M Agany, Bichar Dip Shrestha Gurung, Valena Doe, Zelaikha Yosufzai, Daniel Pan, Ross Campbell, Victor C Huber, Rajesh Sani, Venkataramana Gadhamshetty, Carol Lushbough

**Affiliations:** Biomedical Engineering Department, University of South Dakota, 4800 N. Career Ave., Suite 221, Sioux Falls, South Dakota, 57107, United States; Biomedical Engineering Department, University of South Dakota, 4800 N. Career Ave., Suite 221, Sioux Falls, South Dakota, 57107, United States; Biomedical Engineering Department, University of South Dakota, 4800 N. Career Ave., Suite 221, Sioux Falls, South Dakota, 57107, United States; Biomedical Engineering Department, University of South Dakota, 4800 N. Career Ave., Suite 221, Sioux Falls, South Dakota, 57107, United States; Biomedical Engineering Department, University of South Dakota, 4800 N. Career Ave., Suite 221, Sioux Falls, South Dakota, 57107, United States; Biomedical Engineering Department, University of South Dakota, 4800 N. Career Ave., Suite 221, Sioux Falls, South Dakota, 57107, United States; Biomedical Engineering Department, University of South Dakota, 4800 N. Career Ave., Suite 221, Sioux Falls, South Dakota, 57107, United States; Biomedical Engineering Department, University of South Dakota, 4800 N. Career Ave., Suite 221, Sioux Falls, South Dakota, 57107, United States; Google Cloud, 1900 Reston Metro Plaza, Reston, Virginia, 20190, United States; Health Data and AI, Deloitte Consulting LLP, 1919 N Lynn St., Suite 1500, Arlington, Virginia, 22209, United States; Health Data and AI, Deloitte Consulting LLP, 1919 N Lynn St., Suite 1500, Arlington, Virginia, 22209, United States; Health Data and AI, Deloitte Consulting LLP, 1919 N Lynn St., Suite 1500, Arlington, Virginia, 22209, United States; Basic Biomedical Sciences Division, University of South Dakota, 414 E. Clark St, Vermillion, South Dakota, 57069, United States; South Dakota School of Mines & Technology, 501 E. Saint Joseph St., Rapid City, South Dakota, 57701, United States; South Dakota School of Mines & Technology, 501 E. Saint Joseph St., Rapid City, South Dakota, 57701, United States; Biomedical Engineering Department, University of South Dakota, 4800 N. Career Ave., Suite 221, Sioux Falls, South Dakota, 57107, United States

**Keywords:** metagenomics, quorum sensing proteins, Google Cloud Platform (GCP), Docker, microbe biofilm marker, interactive bioinformatics workflow

## Abstract

In an environment, microbes often work in communities to achieve most of their essential functions, including the production of essential nutrients. Microbial biofilms are communities of microbes that attach to a nonliving or living surface by embedding themselves into a self-secreted matrix of extracellular polymeric substances. These communities work together to enhance their colonization of surfaces, produce essential nutrients, and achieve their essential functions for growth and survival. They often consist of diverse microbes including bacteria, viruses, and fungi. Biofilms play a critical role in influencing plant phenotypes and human microbial infections. Understanding how these biofilms impact plant health, human health, and the environment is important for analyzing genotype–phenotype-driven rule-of-life functions. Such fundamental knowledge can be used to precisely control the growth of biofilms on a given surface. Metagenomics is a powerful tool for analyzing biofilm genomes through function-based gene and protein sequence identification (functional metagenomics) and sequence-based function identification (sequence metagenomics). Metagenomic sequencing enables a comprehensive sampling of all genes in all organisms present within a biofilm sample. However, the complexity of biofilm metagenomic study warrants the increasing need to follow the Findability, Accessibility, Interoperability, and Reusable (FAIR) Guiding Principles for scientific data management. This will ensure that scientific findings can be more easily validated by the research community. This study proposes a dockerized, self-learning bioinformatics workflow to increase the community adoption of metagenomics toolkits in a metagenomics and meta-transcriptomics investigation. Our biofilm metagenomics workflow self-learning module includes integrated learning resources with an interactive dockerized workflow. This module will allow learners to analyze resources that are beneficial for aggregating knowledge about biofilm marker genes, proteins, and metabolic pathways as they define the composition of specific microbial communities. Cloud and dockerized technology can allow novice learners—even those with minimal knowledge in computer science—to use complicated bioinformatics tools. Our cloud-based, dockerized workflow splits biofilm microbiome metagenomics analyses into four easy-to-follow submodules. A variety of tools are built into each submodule. As students navigate these submodules, they learn about each tool used to accomplish the task. The downstream analysis is conducted using processed data obtained from online resources or raw data processed via Nextflow pipelines. This analysis takes place within Vertex AI’s Jupyter notebook instance with R and Python kernels. Subsequently, results are stored and visualized in Google Cloud storage buckets, alleviating the computational burden on local resources. The result is a comprehensive tutorial that guides bioinformaticians of any skill level through the entire workflow. It enables them to comprehend and implement the necessary processes involved in this integrated workflow from start to finish.

This manuscript describes the development of a resource module that is part of a learning platform named ”NIGMS Sandbox for Cloud-based Learning” https://github.com/NIGMS/NIGMS-Sandbox. The overall genesis of the Sandbox is described in the editorial NIGMS Sandbox [1] at the beginning of this Supplement. This module delivers learning materials on the analysis of bulk and single-cell ATAC-seq data in an interactive format that uses appropriate cloud resources for data access and analyses.

## Introduction

**Figure 1:**
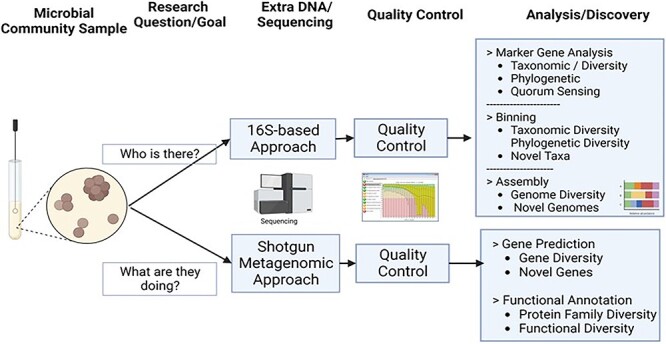
The process of metagenomic analysis, from microbial community sampling to analysis and discovery.

Biofilms are microbial communities comprised of diverse microorganisms such as bacteria, viruses, and fungi. They cause nearly 75% of human microbial infections [1]. Understanding the role of biofilms in human health and how they can be controlled is becoming increasingly relevant for preventive medicine and for managing chronic diseases effectively [2]. Although they dominate our biosphere, most biofilms’ microorganisms have not been cultured; therefore, there is not much information available about their genetic makeup [3]. Broadly speaking, Multi-omics (or integrative omics) in which metagenomics approach is part-taking has provided critical insights into microbial communities and their underlying biological mechanisms [4]. Multi-omics is a powerful technology for understanding interactions between genotypes, environments, and life. This approach can provide researchers with a greater understanding of the flow of information, from the original cause of disease (genetic, environmental, or developmental) to the relevant interactions that yield functional consequences [5]. For example, omics enabled by high-throughput experiments provides researchers with a tool to explore antibiotic resistance and heavy metals through large-scale analysis of multi-omics data. Multi-omics high-throughput data analysis enables an integrative approach to major omics technologies, including nucleic acid-based (genomics, epigenomics, metagenomics, and transcriptomics) and mass spectrometry measurements (proteomics, metabolomics, and fluxomics). Thus, multi-omics makes it possible to characterize in-depth molecular mechanisms of interactions among genes, proteins, and metabolites. This technology improves our understanding of the basic underlying biology and systems-level and physiological processes using a comprehensive approach to investigate human health systems [6]. This high-throughput approach enables scientists to unravel complex multifactorial disease-resistant biofilms through large-scale data analysis of omics data. This domain remains a data-driven enterprise relying mainly on safe, scalable computing with a holistic network infrastructure (see Fig. 1).

Modern bioinformatics pipelines bridge the gap between biological knowledge and implementation. However, they require collaboration among various tools and data sources, which can be challenging for beginners. With efficient computational platforms such as Google Cloud Platform (GCP) and dockerized technology, life sciences students can use complicated bioinformatics tools without much computer science knowledge. Our cloud-based, dockerized workflow is tailored for metagenomics analysis. As an example, it used a biofilm microbiome to showcase the modules that form the workflow. It splits biofilm microbiome metagenomics analysis into five easy-to-follow submodule tutorials. Each submodule is built using various tools, and as students navigate the submodule, they learn about each task and the tools used to accomplish the task.

## Biofilm metagenomics analysis workflow overview

Our metagenomics workflow includes analysis of a biofilm’s composition, diversity, and function. One of the primary objectives of the workflow is characterizing the taxonomic diversity of biofilm communities. This analysis helps provide insights into microbial diversity by identifying and associating specific organisms or taxonomic groups with phenotypic/functional traits characterizing a given environment [7]. Taxonomic classification is challenging because the volume of metagenomics data can be large, yielding high demands on bioinformatics tools. Additionally, genome query sequences of most microbes lack taxonomically-related sequences in existing reference databases [7]. Taxonomic binning is the process of assigning taxonomic identifiers to sequence fragments based on sequence similarity and composition. It is used to draft genome reconstruction. The outcome of the binning process can be used for taxonomic diversity assessment. It can also be leveraged for genome assembly and evaluation of gene association with different taxonomies [8].

Gene prediction and functional annotation are also critical steps in the biofilm metagenomics workflow. Functional annotation of shotgun metagenomic data has become an increasingly popular method for identifying the aggregate functional capacities encoded by the community’s biofilm [9]. This analysis relies on comparisons of predicted genes with existing, previously annotated sequences. Functional profiling provides insights into what functions are carried out by given biofilm communities.

**Figure 2:**
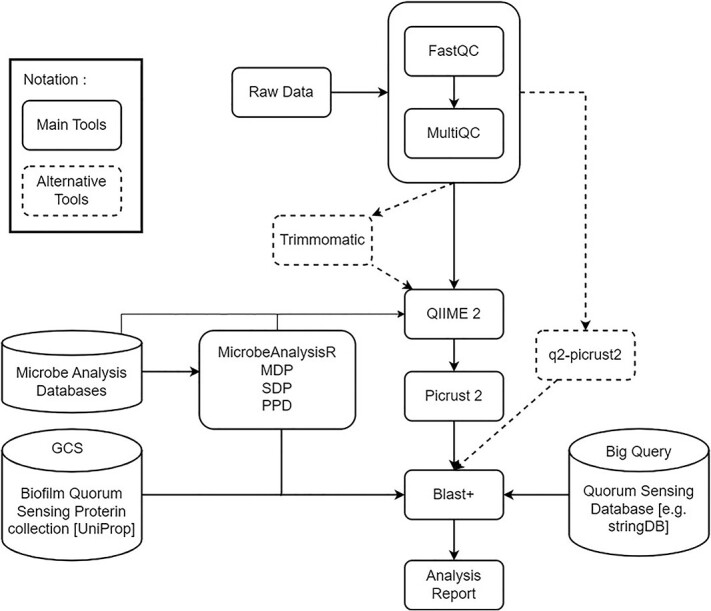
The metagenomics workflow includes the analysis of the biofilm composition, diversity, and function.

Our metagenomics workflow includes the analysis of biofilm composition, diversity, and function. The workflow consists of five submodules and includes the following tools: Docker, Jupyter Notebook, Python custom scripts, FastQC, MultiQC, Trimmomatic, QIIME 2, PICRUSt2, Google Big Query, and BLAST+ (see Fig. 2). Docker is a container platform designed to simplify the creation, deployment, and execution of workflows using containers [10]. Jupyter Notebook serves as a browser-based, open-source platform acting as a virtual laboratory notebook. It aids in managing workflows, code, data, and visualizations, effectively documenting the research process [11]. FastQC is a widely adopted tool that summarizes nucleotide sequence read quality by position, informs the user of adapter content in sequences, and reports on tetramer frequencies [12]. MultiQC is a tool designed to summarize analysis results for multiple tools and samples in a report [13]. Trimming and validating sequence short reads is accomplished using Trimmomatic as an alternative for user practice [14]. The Quantitative Insights Into Microbial Ecology version 2 (QIIME 2) tool is used to transform raw sequences into taxonomic visualizations of microbial diversity [15]. PICRUSt2 was developed to predict the functional potential of a bacterial community on the basis of marker gene sequencing profiles [16]. BigQuery is a data warehouse product managed by Google, designed to be highly scalable, fast, and optimized for data analytics. It also includes rudimentary in-built machine learning capabilities as part of its product offering [17]. BLAST+ is a sequence alignment program that is a central application in bioinformatics [18]. It is important to note that these tools are available in our docker, and users can choose on each step the most relevant tools. For example, with the PICRUSt2 plugin embedded into QIIME 2, a user may choose to run a stand-alone PICRUSt2 for comparative analysis and/or for more flexibility.

**Figure 3:**
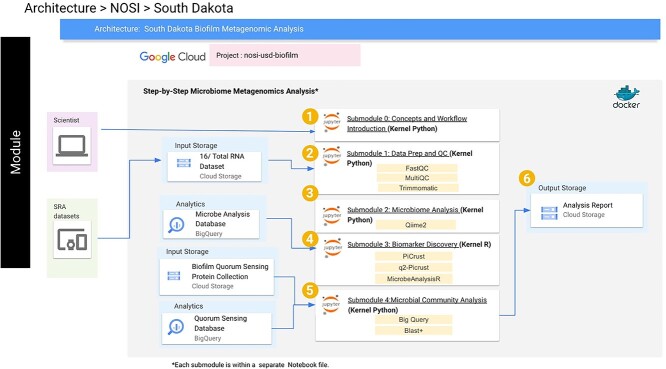
The technical infrastructure diagram of the step-by-step workflow implementation.

The biofilm metagenomics workflow self-learning module is implemented through one introductory submodule and four analytic submodules (Fig. 3). These submodules are titled: Concept Inventory and Workflow Introduction, Metagenome Data Preparation and QC, Microbiome Analysis, Biomarker Discovery, Microbiome Community Analysis, and Metagenomics Analysis of Microbiome Community and Biofilm. A future, sixth submodule will use NextFlow to scale the entire workflow.

A cloud-based approach provides easy access to suitable computational capabilities, but it is important to balance the allocation of the computational services of the analysis workflow in a cost-effective way. In Fig. 3, we show the technical infrastructure diagram of the step-by-step workflow implementation.

## Biofilm metagenomics analysis workflow learning objectives

The biofilm metagenomics workflow self-learning module caters to learners ranging from undergraduate to graduate training level, with learning objectives varying slightly based on the audience (see Table 1). This self-learning module will equip the learner with fundamental concepts related to omics, microbiomes, and biofilm analysis. Upon completion, learners will be proficient in describing and manipulating datasets and toolkits essential for microbiome analysis projects. To assist the learner, we reorganized the full list of Input and Output information for this workflow in our supplementary data at (Ref).

**Table 1:** Biofilm metagenomics analysis workflow learning objectives

**Statement 1:** Biofilms have great importance for public health because of their role in certain infectious diseases and their importance in a variety of device-related infections.		
**Objective:** concept inventory	**Audience**	**Required**
• Describe in general terms ”what is biofilm”	All	Yes
• Microbes and taxonomy/phylogenetic analysis	All	Yes
• Explain why the understanding of biofilm is important	All	Yes
• Demonstrate how to sequence biofilm using the 16S rRNA gene approach	Graduate students	No
• Demonstrate how to sequence biofilm using shotgun metagenomic sequencing	Graduate students	No
• Compare the 16S rRNA and shotgun sequencing approaches	All	Yes
• Explain what is meant by metagenomics	All	Yes
• Explain what is expected from biofilm metagenomics analysis	All	Yes
**Statement 2:** Biofilm metagenomic analysis can be leveraged to aid in our understanding of microbial taxonomy, functions, interactions, ecology, and evolution.		
**Objective:** bioinformatics workflow design and implementation	**Audience**	**Required**
• Explain the input into the biofilm metagenomics workflow	All	Yes
• Explain the importance of each step in the biofilm metagenomics workflow	All	Yes
• Demonstrate how to modify the parameters in the biofilm metagenomics workflow	Graduate students	Yes
• Demonstrate how to change the input into the biofilm metagenomics workflow	Graduate students	Yes
• Demonstrate how to execute the biofilm metagenomics workflow	All	Yes
• Explain the output of the biofilm metagenomics workflow	All	Yes
**Statement 3:** A cloud-based approach provides easy access to suitable computational capabilities, but it is important to balance the allocation of the computational services of the analysis workflow in a cost-effective way.		
**Objective:** bioinformatics workflow design and implementation	**Audience**	**Required**
• Explain FAIR data principles	All	Yes
• Demonstrate how to download the dockerized biofilm metagenomics workflow to a local or other alternate environment,	All	No
• Demonstrate how to customize the biofilm metagenomics workflow	Graduate students	No

## Biofilm metagenomics analysis workflow setup for GCP

The GCP provides an extensive array of services dedicated to safeguarding, storing, delivering, and examining data. These cloud-based solutions establish a secure data perimeter, enabling various operations and transformations to be performed on the data entirely within the confines of the cloud environment [19]. To begin this tutorial, students must set up their GCP, Vertex AI, and Jupyter Notebook instance. To obtain instructions on how to create a Google Cloud account, navigate to the NIH Cloud Lab page and open the README.md file. Next, follow the instructions to set up a Vertex AI Jupyter Notebook and clone the NIGMS Sandbox GitHub repository, which will give access to the modules in Jupyter Notebook.

## Submodule 1: metagenome data preparation and quality control

### Background and biomedical use case

Our learning module introduces the learner to diverse datasets encompassing current state-of-the-art data acquisition techniques in microbial community analyses. In this paper, the data we are using as input into our example analysis for this self-learning tutorial are based on the work done by Gregory *et al*. [20] in their ”Moving Pictures of the Human Microbiome” research. It is available at https://docs.qiime2.org/2023.5/tutorials/moving-pictures/. To advance treatments for potential microbiome-related conditions like obesity, Crohn’s disease, inflammatory bowel disease, and malnutrition, it is vital to comprehend the natural temporal fluctuations in the human microbiome. Nevertheless, sequencing and computational technologies have posed challenges in conducting comprehensive time series analyses on the human microbiome. In their study, Gregory *et al*. [20] address this limitation by presenting the most extensive human microbiota time series analysis conducted to date. The research encompasses data from two individuals, collected at four body sites, and spans 396 time points. We chose this dataset to evaluate the biofilm signature that was not investigated in the study.

### Dataset overview

In the ”Moving Pictures of the Human Microbiome” study, two healthy subjects, one male (M3) and one female (F4)—where the male participated in an earlier survey [21]—were sampled daily at three body sites (gut (feces), mouth, and skin (left and right palms)), for 15 months (M3) and for 6 months (F4) using an institutional review board-approved protocol [20]. To streamline the massively parallel sequencing of 1967 samples, the identical barcodes were utilized across six lanes within a single Illumina GAIIx instrument. The sample distribution across these lanes was as follows: 374, 372, 364, 271, 265, and 323 samples in lanes 1 through 6, respectively. All sequence data and sample metadata are publicly available under the ”Moving Pictures of the Human Microbiome” project [MG-RAST:4457768.3-4459735.3], and these data were downloaded for this module.

### Methods and results

FastQC and MultiQC are executed to evaluate the raw dataset quality. Each Jupyter Notebook cell focuses on one dataset and creates an output directory to store each quality control report. At the completion of this step, an output folder containing multiple files and a navigable HTML file to visualize the data set quality is created. Several interactive figures are generated, including the ”per base quality score” example maps that report whether these reads need to be trimmed to remove adapter sequences and low-quality bases.

In summary, Submodule #1 walks the student through using data preparation and quality control checks with the tools FASTQC and MultiQC. The student also learns how to trim poor-quality reads.

## Submodule 2: microbiome analysis

### Background and biomedical use case

One of the workflow’s primary objectives is to characterize the taxonomic diversity of biofilm communities from 16S data using QIIME 2. This step helps provide insights into microbial diversity by identifying and associating specific organisms or taxonomic groups with phenotypic/functional traits characterizing a given environment. Taxonomic classification is challenging because the volume of metagenomics data is large and puts high demands on computational resources. Additionally, queried sequences of most microbes lack taxonomically related sequences in existing reference databases. Taxonomic binning—the process of assigning taxonomic identifiers to sequence fragments based on sequence similarity and composition—is used to reconstruct draft genomes [22]. The outcome of the binning process can then be used not only for taxonomic diversity assessment but also for genome assembly and evaluation of gene association across different taxonomies.

### Dataset overview

The following script is from the QIIME 2 tutorial workshop [23] that looks into two human samples at four different body sites. Typically, when running a metagenomic analysis, what is in the sample may not be known. To help identify them, sequences are tagged with barcodes that can be looked up in a corresponding metadata table alongside other relevant information (e.g. location collected, data, time, sample source, etc.). In all, there are three sets of inputs:

- Sequence file: Contains all the sequences found in the sample.
- Barcode file: Contains all the barcodes with which each sequence is tagged.For example, the Illumina’s i5 and 17 barcodes.
- Metadata file: This file contains sample information and each sample’s barcode. Ours holds the following information: the body site where the sample was collected, the sample ID, and the time and date the sample was collected.

### Methods and results

The QIIME 2 script consists of multiple analytic and visualization steps. Detecting and eliminating multiplets plays a crucial role in enhancing the scalability and dependability of metagenomics sequencing [24]. The “demux” function demultiplexes the sequences to identify which barcode is associated with each sample, which will, in turn, help to identify which sequences are associated with each sample. DADA2 identifies and filters out any phiX and chimeric sequences. In other words, it will trim any low-quality reads [25]. The result will produce a feature table containing counts of each unique sequence in each sample within our dataset and feature data that map feature identifiers in the feature table to the sequences they represent. This module utilizes core-metrics-phylogenetics to compute alpha and beta diversity metrics and alpha-group-significance tests to assess the associations between categorical metadata columns and alpha diversity data. Additionally, beta-group-significance tests will be employed to analyze the relationship between beta diversity data and the same categorical metadata columns. EMPeror generates principal coordinate (PCoA) plots using distance matrices obtained from beta diversity analysis. These plots effectively illustrate the relationships between samples by representing their microbiome profile similarities. Feature-classifier will predict the taxonomic composition of the samples in the FeatureData file based on a pre-trained Naive Bayes classifier that we have provided.

The “feature-table filter-samples” command performs a specialized differential abundance analysis using an Analysis of Composition of Microbiomes (ANCOM). This analysis focuses on identifying features that exhibit differential abundance across gut-associated sample groups. This step involves several sub-processes to ensure a comprehensive examination, which includes creating a feature table that contains only the gut samples and a feature count table. ANCOM is executed to identify the difference in abundance based on the features from our metadata between the two subjects.

This module includes QIIME 2 commands that generate visualizations for nearly every step, allowing users to view these visuals directly in the Jupyter notebook. At the completion of our QIIME 2 analysis and post-processing, all output is uncompressed in QIIME 2’s custom output format. This module allows for the extraction of content and access to readable formats such as HTML, comma-separated values (.csv), and figures in our “qiime2_Output” directory. Any directory with the notation “viz”** contains visuals such as plots and figures that can be explored.

In summary, Submodule #2 guides students through analyzing sequencing datasets and mapping reads to relevant microbiome taxonomy. The student also learns to retrieve and understand the microbiome community available on the input dataset (alpha and beta diversity). Lastly, the student learns how to interpret microbiome analysis results using Qiime2.

## Submodule 3: biomarker discovery

### Background and discovery goal

In the biofilm metagenomics workflow, microbiome community gene prediction and functional annotation are crucial steps. Functional annotation of shotgun metagenomes data has gained popularity as a means of identifying the collective functional capabilities encoded by the biofilm community. This analysis involves comparing predicted genes with previously annotated sequences from 16S and/or metagenomes samples, providing insights into the functions performed by the biofilm community.

PICRUSt2 is the primary tool for functional annotation of 16S metagenomes data. It can be utilized as a standalone tool, integrated as a QIIME 2 plugin, or employed through the MicrobeAnalystR wrapper workflow. On the other hand, the QIIME 2 shotgun module or other tools are used for both taxonomics and function annotation of shotgun metagenome data. In this submodule, we showcase examples of each approach.

### Dataset overview

PICRUSt2 utilizes machine learning to forecast the functional abundance and capabilities of microbial communities using 16S rRNA marker genes. To initiate the analysis, we will define the environment by indicating the location of PICRUSt2 inputs and outputs. This practice streamlines file tracking and eliminates the need for redundant path typing. Initially, PICRUSt2 is executed as a standalone tool, simplifying the process by defining specific data paths as environment variables. This allows the PICRUSt2 scripts to automatically find the required data without manual intervention.

The input “fasta” and “biom” files are both outputs from denoise analysis using DADA2 in Submodule #2. To break it down:

- The FeatureData is the “fasta” file (also written as “fna”) and contains amplicon sequence variants (ASV) of 16S rRNA reads and IDs found across the human samples.
- The FeatureTable is our “biom” file that contains the IDs of the ASV reads and the number of times these reads were found per sample.

Next, the environment variables are set with the number of available cores on this virtual machine. Since the number of cores will change with each machine type, it is important to capture this with a variable rather than pass a hard-coded integer as an argument for each multi-threaded step.

### Methods and results

The PICRUSt2 pipeline includes several scripts. The “place:seqs.py” script inserts the ASVs reads into a reference tree based on the Integrated Microbial Genomes database (https://github-wiki-see.page/m/picrust/picrust2/wiki/PICRUSt2-Tutorial). This produces the output tree file that will be our input for the next command. The “hsp.py” script predicts the copy number of gene families for each ASV. The script is run twice in order to identify sequences with the 16S rRNA marker and their Enzyme Classification (EC) number. The “metagenome_pipeline.py” script does the same thing as the “hsp.py” script but the difference is that it predicts gene families weighted by the relative abundance of ASVs in their community.

The output should show that some sequences are above max nearest-sequenced taxon index (NSTI) cut-off of 2.0. The NSTI is the branch length between the nearest 16S reference sequence and each ASV. The presumption is that as the NSTI value decreases, the closer the relationship is between the ASV reads and the corresponding 16S sequence. Anything above 2.0 is considered noise and will not be used in the analysis. The findings are summarized creating visuals, histograms, and statistics on how many sequences are associated with each sample and feature. Non-phylogenetic diversity metrics and feature tables are also created.

In summary, Submodule #3 teaches students how to extract microbiome biomarkers using several computational tools. They learn how to use QIIME2 outputs to predict relevant proteins and pathways from a 16s dataset using a PICRUSt2 pre-trained machine learning model. For a shotgun-generated dataset, they learn how to use the QIIME 2 shotgun module for both taxonomy and functional profiling or downstream systems biology analysis.

## Submodule 4: microbiome community analysis for biofilm marker identification

### Background and marker identification goal

In microbial community analysis, comparisons are made between predicted genes, proteins, functions, and previously annotated sequences. Functional profiling provides valuable insights into the functions performed by the microbial community. Our current workflow does not have a biofilm signature assessment; this limits the ability to identify relevant biofilm markers in a study. Quorum sensing (QS) serves as a crucial indicator of bacterial community behavior with strong relation to biofilm formation [26]. QS involves the regulation of gene expression in response to changes in cell-population density. Bacteria employing QS release chemical signaling molecules called autoinducers, whose concentration increases with cell density. Although the presence of QS signaling does not guarantee biofilm formation, it has proven to be a reliable marker in various phenotype analyses of biofilms, including those associated with cancer, dental health, medical devices, corrosion, and environmental biofilms. Upon completion of Submodule #4, students are able to extract relevant candidate markers of biofilm formation in each sample.

### Dataset overview

To carry out the community analysis for biofilm marker identification, protein names of each EC number are retrieved from the “pred_metagenome_unstrat.tsv” output that was generated by the PICRUSt2 analysis from Submodule #3. Each EC number is extracted and searched against the UniProt protein database. All outputs are then added to a protein names list. It is important to note that our workflow will use other datasets pre-loaded into Google BigQuery to facilitate the user experience as described below.

### Method and results

In this module, we utilize the STRING Database [27] and BLAST+ to search for biofilm signatures within our metagenomes samples. The protein names list is queried against the STRING Database that NIH has hosted as a public BigQuery database. An alternative database that can be used is the Biofilms Structural Database (BSD), a collection of structural, mutagenesis, kinetics, and inhibition data to understand the processes involved in biofilm formation [28]. Two queries are executed: the first to collect the protein IDs associated with the names retrieved from the UniProt database, and the second to extract the sequences that are associated with the protein IDs. Both are required in the BLAST+ execution. BLAST+ (specifically BLASTp) allows for the querying of multiple protein sequences through the BLAST database and retrieves a collection of hits that includes protein IDs with sequences similar to our collection. The code performs the following steps:

1. Makes a custom BLAST database based on our pre-made “fasta” file from UniprotKB and creates our quorum sensing protein collection.
2. Queries our own “fasta” file against the custom database to see if we have any plausible hits.
3. Adds headers to our output file to better understand the results.

These commands are executed via Google Batch. This feature allows us to run our BLAST search in parallel by starting up multiple virtual machines. Each machine runs a different job, allowing us to receive our output faster. We want e-values that are very close to 0 because it tells us our hit is very significant. With these results we can conclude that there are biofilm markers within our dataset.

In summary, Submodule #4 teaches how to perform microbial community and biofilm analysis. The student learns how to use Google BigQuery with the STRING database. They also are guided through how to run BLAST+ through Life Sciences API and how to analyze the results.

**Key Points**

- This paper proposes an easy-to-use, cloud-based dockerized workflow for Biofilm Metagenomics analysis and marker prediction.
- The proposed dockerized, self-learning bioinformatics workflow aims to facilitate the adoption of metagenomics toolkits in investigations.
- The integrated learning resources and interactive modules allow learners, regardless of their computer science knowledge, to analyze biofilm marker genes, proteins, and metabolic pathways.
- Leveraging cloud and dockerized technology, the workflow simplifies biofilm microbiome metagenomics analyses using Vertex AI’s Jupyter notebook instance, with results stored and visualized in Google Cloud storage buckets.
- This comprehensive tutorial is designed to guide bioinformaticians of all skill levels through the entire workflow, making it accessible and implementable.

## Data Availability

The code and datasets of Metagenomics Analysis of Biofilm Microbiome are available on GitHub (https://github.com/NIG MS/Metagenomics-Analysis-of-Biofilm-Microbiome). The repository’s FigShare DOI is 10.6084/m9.figshare.25408411.v1 and can be accessed at https://figshare.com/articles/software/Metagenomics-Analysis-of-Biofilm-Microbiome/25408411. A FAIRshake assessment of our workflow can be found at https://fairshake.cloud/digital_object/813126/assessments.

## Conclusion and future work

These modules provide researchers with an easy-to-execute metagenomics self-learning workflow that includes analysis of biofilm composition, diversity, and function. These modules are running on GCP and powered by Google, Deloitte, and NIH teams. Our workflow aims to introduce the learner to microbiome analysis through a self-paced practical learning module that will aid in the understanding of the role of biofilms in animal health plant health, and even events such as biocorrosion. Our module includes the analysis of microbial community composition (e.g. what microorganisms are in the sample?), diversity, and function (e.g. what are they doing?). We leverage quorum sensing protein signatures to provide insights into the microbial community and biofilm gene–phenotype response marker prediction.

Our future goal will leverage the biofilm community’s efforts to evaluate and update the workflow to capture new multi-omics and meta-omics data modalities such as meta-transcriptomics and meta-proteomics to empower the relevance of our predicted biofilm markers. This effort will also allow us to scale the workflow and its implementation to include Nextflow (under development), and expand the framework to run on other cloud platforms such AWS and Azure.
